# Midwives’ respect and disrespect of women during facility-based childbirth in urban Tanzania: a qualitative study

**DOI:** 10.1186/s12978-017-0447-6

**Published:** 2018-01-10

**Authors:** Kana Shimoda, Shigeko Horiuchi, Sebalda Leshabari, Yoko Shimpuku

**Affiliations:** 1 St. Luke’s International University, 10-1 Akashi-cho, Chuo-ku, Tokyo, 104-0044 Japan; 2 St. Luke’s Birth Clinic, 1-24 Akashi-cho, Chuo-ku, Tokyo, 104-0044 Japan; 3 School of Nursing, Muhimbili University of Health and Allied Sciences, P.O. Box 65004, Dar es Salaam, Tanzania

**Keywords:** Disrespect and abuse, Mistreatment, Quality of care, Facility-based childbirth, Respectful care, Humanized childbirth, Tanzania

## Abstract

**Background:**

Over the last two decades, facility-based childbirths in Tanzania have only minimally increased by 10% partly because of healthcare providers’ disrespect and abuse (D&A) of women during childbirth. Although numerous studies have substantiated women’s experience of D&A during childbirth by healthcare providers, few have focused on how D&A occurred during the midwives’ actual care. This study aimed to describe from actual observations the respectful and disrespectful care received by women from midwives during their labor period in two hospitals in urban Tanzania.

**Methods:**

This descriptive qualitative study involved naturalistic observation of two health facilities in urban Tanzania. Fourteen midwives were purposively recruited for the one-on-one shadowing of their care of 24 women in labor from admission to the fourth stage of labor. Observations of their midwifery care were analyzed using content analysis.

**Results:**

All the 14 midwives showed both respectful and disrespectful care and some practices that have not been explicated in previous reports of women’s experiences. For respectful care, five categories were identified: 1) positive interactions between midwives and women, 2) respect for women’s privacy, 3) provision of safe and timely midwifery care for delivery, 4) active engagement in women’s labor process, and 5) encouragement of the mother-baby relationship. For disrespectful care, five categories were recognized: 1) physical abuse, 2) psychological abuse, 3) non-confidential care, 4) non-consented care, and 5) abandonment of care. Two additional categories emerged from the unprioritized and disorganized nursing and midwifery management: 1) lack of accountability and 2) unethical clinical practices.

**Conclusions:**

Both respectful care and disrespectful care of midwives were observed in the two health facilities in urban Tanzania. Several types of physical and psychological abuse that have not been reported were observed. Weak nursing and midwifery management was found to be a contributor to the D&A of women. To promote respectful care of women, pre-service and in-service trainings, improvement of working conditions and environment, empowering pregnant women, and strengthening health policies are crucial.

## Plain English summary

In recent years, numerous studies around the world have described the disrespect and abuse (D&A) experienced by some women during childbirth from healthcare providers at facilities. In particular, Tanzanian women have experienced physical and verbal abuse, as well as being ignored and neglected when birthing at facilities. Unfortunately, there have been few studies of D&A carried out by direct observations of midwives’ actual care during childbirth. This study aimed to make actual observations and describe the respectful and disrespectful care received by women from midwives during childbirth in urban Tanzania.

Direct observations of the childbirth care provided by the 14 midwives revealed both respectful and disrespectful care. Some midwives had positive interactions with the women, showed respect of their privacy, provided safe and timely care for delivery, actively engaged in the women’s labor process, and encouraged mother-baby relationship. However, some midwives abused the women physically and psychologically, showed no respect of their privacy, failed to obtain consent before giving care, and ignored and neglected the women during their childbirth. Several kinds of physical and psychological abuse that have not been reported were also observed. The unprioritized and disorganized nursing and midwifery management was an important factor contributing to the disrespect for women.

To promote respectful care by midwives, pre-service and in-service trainings, improvement of the working conditions and environment, and strengthening of health policies are crucial. It is also important to not only identify positive approaches to supporting midwives but also empower women to know their rights regarding being treated respectfully.

## Background

Over the last two decades, there has been a global increase in facility-based childbirths resulting from efforts to reduce maternal and infant deaths [1, 2]. However, the percentage of recent childbirths at health facilities in Tanzania has shown only a minimal increase of 10% compared with the 52.6% increase in 1991–1992 and the 63% increase in 2015–2016 [1, 3]. In low-income areas, barriers, such as financial, infrastructural, sociocultural, and political factors have been noted to affect women’s utilization of health facilities for childbirth [4, 5]. Inadequate and unsafe care by healthcare providers including disrespectful, abusive and neglectful care, and the negative experiences of women particularly during childbirth that violate the trust between women and healthcare providers, have also been identified as important contributors to the women’s underutilization of health facilities [6–8].

In this context, a particular concern is the quality of facility-based care during childbirth [9]. Historically, the areas of health coverage and quantity of healthcare providers have been the focus of program implementation at the national level [10]. It has only been recently that the quality of care has received attention [7]. In recent years, however, more studies have reported on women’s experiences of disrespectful and abusive care during childbirth at facilities by healthcare providers. Bowser and Hill (2010) systematically reviewed disrespect and abuse (D&A) by healthcare providers and categorized the various forms of abuse as physical abuse, non-consented care, non-confidential care, non-dignified care, discrimination, abandonment, and detention in facilities [4]. Moreover, these categories may overlap [4, 7] and can occur along a continuum from subtle discrimination to overt violence [11]. In Tanzania, quantitative studies on midwifery have also revealed the negative care experiences of women. Approximately 12 to 70% of women have been found to experience D&A when birthing at facilities [8, 12–16].

These findings have caused policy makers and clinicians to start expressing their growing concern regarding the quality of care provided during childbirth in health facilities in both low-middle income and high-income countries. In 2014, WHO made the following statement: “The prevention and elimination of disrespect and abuse during facility-based childbirth”, which indicated the lack of an internationally agreed definition and measurement tool of D&A and the urgency of the problem [17]. Most international qualitative and quantitative studies on the disrespectful and abusive behaviors of healthcare providers have been based only on women’s reports. Only few studies have focused on how D&A occurred when midwives provide actual care during childbirth. Thus, this study aimed to describe from actual observations the respectful and disrespectful care received by women from midwives during their labor period in two hospitals in urban Tanzania.

## Methods

### Study design

The study design was a qualitative descriptive study using naturalistic observation of midwives during childbirth in the labor wards of two hospitals in urban Tanzania in November and December 2014.

### Settings

The study was conducted at two consenting health institutions whose average monthly numbers of deliveries were 110 and 1800, respectively. Midwives worked in three shifts (morning, evening, and night), and between three to eight midwives covered each antenatal and labor ward during the morning and evening shifts, although there were fewer midwives on the night shifts.

### Sample and recruitment

Purposive sampling was used because only experienced midwives would be able to fulfill the aim of the present research. For the inclusion criteria, the participants should 1) be a nurse-midwife (midwife) and 2) have experienced conducting deliveries for at least one year.

Prior to the participation of the midwives, two research assistants explained the purpose, methods, and ethical considerations of the present study and obtained their consent to participate. Enrollment in the study was conducted based on the principles of voluntarily participation. A poster prepared in Swahili was placed on the labor ward, which included an explanation that the midwife-researcher (herein researcher) was observing the midwives’ actions and was not obtaining personal or medical information from the mothers and babies. When the observation began, the researcher first explained the purpose of the study to the mother to obtain verbal consent, and then started observing only after she agreed. The researcher did not ask any questions and only listened to the complaints of the mothers.

Prior to data collection, we obtained informed consent from 14 midwives (eight from one facility and six from another facility), who met the inclusion criteria. Of these, four were registered midwives who were diploma holders, and 10 were enrolled midwives who had completed a certificate course.

### Data collection

One-on-one shadowing of midwives (naturalistic observation) was conducted in the antenatal and labor wards. The first author (KS; herein “researcher”), a midwife who was trained in naturalistic observation and also had previous experience as a participant observer, made the observations of the midwives. Each midwife was observed once for one cycle as she typically cared for a woman from admission to the fourth stage of labor as well as other women who were complaining of labor pains at the antenatal ward. The observation lasted from two hours until the end of delivery (maximum time of five hours).

To minimize the observer effect, the researcher observed each midwife from a distance and took memos when alone and not in front of the other midwives. The researcher committed to memory what has transpired during the observations. The researcher informally asked the midwife on the scene or after the observation in the following occasions: when questions related to the midwife’s action emerged; when the researcher could not understand the midwife’s intention for doing something; when the researcher wanted to know what the midwife thought and how she made a judgement while doing simultaneous actions. Immediately after completing the observations, the field notes and remarks of the midwives were made as fair copies using an observational guide developed and designed by the researcher. The guide included the date and time of the observations, contents of the observed scene, observed actions and attitudes of the midwives, and the working environment.

### Ethical considerations

In the process of developing the observational protocol, it was realized that the researcher might be placed in a difficult position of observing midwife care that is abusive or dangerous to the patient. An example of this is suturing the perineum without anesthesia. We needed to resolve a priori the balance between the extent of obligation as a midwife to protect the patient and the role of the researcher to observe [18]. Neither the WHO expert working group who reviewed existing international ethical guidelines nor a thorough literature review conducted by WHO staff found studies or guidelines clarifying when or whether there was a duty to intervene [19]. Lacking a license as a Tanzanian nurse, the researcher’s role was clarified as not to take any action even if abusive care is observed and instead choose a neutral stance as a naturalistic observer. After completing the observations, the researcher can share and discuss what she observed with the collaborating midwives and the research institution.

### Data analysis

Content analysis was used to analyze the data [20]. After each observation, the researcher recalled the events and they were integrated into the field notes. The midwives’ remarks were written as a verbatim recording. The field notes and transcripts were read and reread highlighting the words, sentences, and situations that indicated the midwives’ actions that were related to respect and disrespect of women during childbirth. The highlighted descriptions were examined and then grouped into subcategories. The subcategories showing conceptual relation were abstracted into categories. The co-authors, who were leading researchers of maternal health and midwifery, discussed and supervised the data analyses. The third author and research assistants provided quality checks of the analysis based on their deep understanding of Tanzanian culture. Providing credibility [21] to the observations involved the following processes: 1) documenting both positive and negative interactions, 2) accounting for research reflexivity understood as the strengths and weakness of the researcher’s perspective in shaping what data would be observed, and 3) collaboration with Tanzanian researchers.

### Ethical approval

The Ethics Review Board of St. Luke’s International University, Tokyo (approval number: 14–084) and the Tanzanian National Institute of Medical Research approved the study.

## Results

The mean age of the participants was 33.9 years (range 24–42). Their mean number of years of experience as midwives was 7.7 (range 1–19). Three main categories were derived as follows: I) **respect for women**, II) **disrespect of women**, and III) **unprioritized and disorganized nursing and midwifery management**. All 14 midwives observed gave disrespectful and abusive care, although five of them also gave respectful care.

### Respect for women

Throughout the whole process of labor, five midwives consistently attempted to assess the progress of labor and took timely and appropriate procedures for delivery. They usually took care of the women politely and respectfully during the care process.

*Respect for women* was supported by five categories that were extracted from the data: 1) positive interactions between midwives and women, 2) respect for women’s privacy, 3) provision of safe and timely midwifery care for delivery, 4) active engagement in the labor process, and 5) encouragement of the mother-baby relationship.

#### Positive interactions between midwives and women

The midwives gave proper consideration to the women’s emotions and practiced good communication skills by introducing themselves, providing timely advice, and expressing empathy.

*At the examination room, midwife E calmly told the woman to lie on the examination bed. After making sure that the woman was reclining, midwife E went to the woman’s side and introduced herself and told her that she was the midwife who would provide her care and that she would start the examination. (Episode [EP] no. 1).*

The midwives expressed empathy and compassion for the women, especially when they received an invasive medical procedure or suffered from labor pains.

*Midwife F called the woman to the examination room for the doctor’s rounds. Midwife F served as the doctor’s assistant. When the doctor was performing vaginal examination, the woman was screaming because of the pain from the examination. Upon observing the situation, midwife F offered to the woman (in Swahili) the sympathetic comment “I am sorry for you” and also gave her a reassuring smile. (EP no. 2).*

Moreover, when the midwives performed physical examinations or medical treatments, they explained what they were going to do, provided the results, and gave their own assessment and advice.

*Midwife C was talking to the woman while checking her blood pressure. After checking, she told the woman that the measurement “was normal”. During that time, the woman cried because of labor pain. Midwife C discontinued the examination and gently advised the woman on how she could relieve her the pain by imitating the proper breathing technique, namely, “give a short breath like huff, huff”. Then after midwife C completed her questions, she gently told the woman that “the baby would not be delivered very soon”. Midwife C also advised the woman that she had “better walk around rather than lie on the bed, take a cup of tea whenever she wanted, and not to take herbal leaves.” (EP no. 3)*

#### Respect for women’s privacy

Most of the examination areas and labor beds were in rooms readily visible to others, although several beds were partitioned with curtains. Some of the midwives were considerate and protected a woman’s privacy from other women using partitions and clothes.

*Midwife E called a woman over to the admission room. Immediately after the woman entered the room, midwife E closed the door and moved a partition curtain across the door. (EP no. 4)*

#### Provision for safe and timely midwifery care for delivery

Along with the periodic monitoring of the labor process, some midwives performed appropriate care for delivery with precise timing by judging the women’s labor process. The following midwife (midwife K) brought the women to the labor ward at an optimal time for delivery and made timely preparations.

*Midwife K realized that woman C was yelling while she was still in the antenatal ward. Upon hearing the tone of her loud voice, midwife K decided to bring woman C to the labor ward. Midwife K told woman C to lie down on her back and to open her legs with her knees bent. Midwife K wore gloves and applied antiseptic with her previously prepared swab to woman C’s perineum. Soon after the rupture of the membrane, the fetal head was crowning. Midwife K supported her perineum and the fetus was delivered soon after she provided support to the perineum. (EP no.6)*

The following midwife (midwife F) also made the appropriate judgement when to move a woman to the labor ward and which treatment was needed to induce labor progression. When the observing researcher asked why she decided to move the woman to the labor ward, this midwife explained to the observing researcher the reason for providing the nursing actions.

*There was yelling and crying from a woman in the antenatal ward. Midwife F checked her chart and said to the researcher that, “She was fourth gravida, and her cervix was already dilated seven centimeters - she is crying, so I will move her to the labor ward.” Midwife F assessed that “her labor was progressing”. After the woman lay on the delivery bed, midwife F inserted an intravenous line and gave fluids “because she didn’t eat and drink for a long time and she seemed to be tired”. (EP no. 7)*

#### Active engagement in the labor process

Midwives collected both subjective and objective data to grasp and assess the progress of labor. They constantly went to the women’s side and asked how they were feeling so as not to overlook any signs of the progression of labor. Throughout these activities, they attempted to predict what would be expected for the women’s childbirth.

*Woman ‘A’ who was lying on a bed in the antenatal ward was suffering from labor pains. Her uterine contractions occurred every three minutes. Midwife D instructed woman ‘A’ to move to the labor ward. After reaching the labor ward, she complained of increasing labor pain. Midwife D asked, “Are you feeling [the need] to push?” and woman ‘A’ answered, “Yes”. Midwife D asked her to lie on her back and she performed vaginal examination and said, “eight centimeters dilated”. After 30 minutes, midwife D went back to the delivery room to check the condition and progress of labor of woman “A”. (EP no. 8)*

The midwives occasionally judged the progress of labor by the women’s call. When the women called them, they reacted and took actions such as running to the women.

*Woman B was calling “Nurse! Nurse!”. Midwife D who was in the nurses’ station stood up and started listening to the voice, and then went from the nurses’ station into the labor ward. Woman B was lying on her right side on the delivery bed. Midwife D found that woman B’s blood was returning and passing through the intravenous line, and the midwife understood why she was called. After she replaced the empty IV bottle with a new bottle, she asked woman B “how are you feeling and how about the labor pains?” (EP no. 9)*

#### Encouragement of the mother-baby relationship

Before moving to the postnatal ward, the midwives prompted the women to start breastfeeding immediately after giving birth even while they were still in the delivery beds to encourage the mother-baby relationship.

*Midwife K instructed the woman who had just delivered to sit on the edge of the delivery bed. When the woman was seated, midwife K asked the woman to hold her baby in her arms and midwife K encouraged her to start breastfeeding using verbal instructions and gestures. Then, the woman was able to start breastfeeding. (EP no. 5)*

### II. Disrespect of women

Although the midwives treated the women respectfully, they all appeared disrespectful, abusive, and harmful at some points when providing care. This disrespectful treatment was classified into five categories: 1) physical abuse, 2) psychological abuse, 3) non-confidential care, 4) non-consented care, and 5) abandonment of care.

#### Physical abuse

Midwives occasionally used force to compel women’s obedience such as beating, slapping, kicking, or pinching during childbirth.

*Midwife D was staring at woman C silently and waiting for the fetal head that was crowning. When woman C tried to close her legs and turn over in the bed because of the labor pain, midwife D slapped her on the inner side of her thigh and said in a harsh tone, “open!!” (EP no. 10)*

Occasionally, the midwives aggressively caused harm and injured the women by giving inappropriate care and treatment by not following the right procedure as follows: artificial rupture of the membranes using a fragment of broken glass ampule, not following the doctor’s instruction for the oxytocin dosage, or suturing perineal tears without the use of anesthesia.

*Woman J had been suffering from labor pains. Midwife I went to her to see how the labor was progressing. Midwife I explained to the researcher, “the uterus contractions were not strong enough to progress”, [which was why] she looked around and found a broken glass ampule that had been left on the table. She quickly inserted the broken glass ampule into the vagina of woman J. Then, she tried to break the membrane with the cutting edge of the ampule but was not successful in spite of several attempts. She then gave up, left woman J, and returned to the nurses’ station. (EP no. 11)*

Despite the exact dosage instructions for oxytocin, some of the midwives did not follow the instructions and they administered a dose that increased the risk for dangerously strong uterine contractions.

*Midwife L received the prescription and order from a doctor to administer oxytocin to woman K. The infusion rate and dose escalation, including the dose increment between the time intervals were written on the prescription. However, midwife L started the IV drip without minding the infusion rate or even using a watch to monitor the drip rate. (EP no. 12)*

Some of the midwives were not concerned whether the women suffered from pain during the suturing of perineal tears; therefore, they did not use any anesthesia.

*Midwife I brought the needle holder, needle, and thread from the other room, and started to stitch the woman’s perineum tear resulting from a delivery without using any anesthesia. The woman screamed to complain about pain, but midwife I continued to stitch while ignoring the woman’s screams. (EP no. 13)*

#### Psychological abuse

The midwives used not only physical force but also psychological force which included emotional and mental abuse in the forms of berating, threatening, and intimidating women and having no consideration for the women’s situation.

*Woman D was vomiting. Midwife B found that there were contaminants mixed in the contents of the vomitus and realized that woman D took some traditional herbal medicine believed to strengthen uterine contraction and promote smooth labor. Midwife D scolded her in harsh tones for taking the herbal medicine, “How many times were you told not to take the local herb?” Other midwives also joined in by berating woman D and began exclaiming: “Why did you take it?” “Who gave it to you?” “Your baby will die if you take it!” (EP no. 14)*

*Woman E was lying on her back in the delivery bed and yelling. Midwife M went to her because she heard her screaming. Midwife M stood and rose to her full height at her bedside and lambasted her saying, “Push enough! Push more strongly!” Woman E was writhing and crying. Midwife M threatened her, “Don’t cry, or your baby will die!” (EP no. 15)*

It is appalling that despite the women’s suffering from their labor pain, the midwives failed to provide soothing relief or full support. They also failed to provide physical support such as touching or emotional support such as sympathetic comments.

*While woman F was walking from the antenatal ward to the labor ward under agonizing labor pains, midwife D was just silently standing in the labor ward with her hands on her hips just watching woman F walking. Woman F stopped many times to hold herself up during the labor pains, but midwife D never went near her or say anything to her. Midwife D instead concentrated on preparing the bed and slowly donned gloves while chatting with other staff. (EP no. 16)*

Sadly, only a few midwives attended to the women’s sorrow or celebration. Even when some women lost their babies, the midwives just cleaned the facilities with no apparent empathy or commiserating words of sympathy or condolence.

*Woman G delivered a stillbirth baby. Midwife I, who came in just before the baby was coming out, pulled the baby out and just put the baby between the legs of woman G. After midwife I recognized that the baby was not breathing, she just gave the back of the baby some taps as an attempt to resuscitate, but she quickly slowed down and stopped her attempts after seeing that the baby did not respond. Midwife I casually told women G, “your baby is dead”, and then she wrapped and took the baby to the sanitary room without even allowing the mother to hold her baby. Woman G just stared at the ceiling and looked vacantly into space. After midwife I returned, she let woman G stand up but said nothing to her. (EP no. 17)*

#### Non-confidential care

It was quite common that midwives would just invade the women’s physical and psychological privacy. As both the antenatal and labor wards were shared rooms, the women could be easily seen or heard by others because there was no partition. Moreover, the treatments administered by the midwives could also be readily seen. Occasionally, the midwives asked the women their private or personal information in front of others.

*Woman A was lying on the bed in the antenatal ward agonizing from labor pains and was yelling for help. When midwife A realized that woman A was yelling, midwife A shouted at woman A in the labor ward saying “Who is yelling?” Midwife A then noticed woman A who was bearing the brunt of the pain and she shouted at woman A again in front of all the other women asking “What is your name?”, “How old are you?”, “How many times have you given birth?” (EP no. 18)*

#### Non-consented care

When the midwives needed to perform a medical treatment or physical examination, they often performed the procedure suddenly without any explanation or consent from the women.

*Midwife K was standing in front of woman B who was lying on a delivery bed. Midwife K just suddenly instructed her to remove the sheet covering her lower half and to spread her legs widely without any explanation. Next, midwife K began to silently clean woman B’s perineum. After cleaning, she picked up the clamps and quickly inserted the tip into woman B’s vagina to break the membrane. Woman B flinched but said nothing. (EP no. 19)*

#### Abandonment of care

At some point, most of the midwives were observed ignoring, neglecting or abandoning women during childbirth. They did not show any concern for the women’s suffering despite their yelling for help. Consequently, many deliveries were conducted without the benefit of midwives’ care.

*Woman H was screaming loudly in the labor ward and calling the midwives saying “Ahhhhhhhhh!! Nurse!! Nurse!!”. Midwife N was sitting and chatting with other staff at the nurses’ station, which is not far from the labor ward. (Since the labor ward opens into the nurses’ station, they can hear the women’s voices even at the nurses’ station.) After a while, midwife N peeked at woman H and said, “Don’t sit like that! Just lay on the bed, but don’t do anything!” Afterward, woman H continued yelling and calling the midwives. Her yelling gradually became loud, but midwife N was taking a nap face down on the desk at the nurses’ station. Eventually, the woman screamed, “please!! please!! coming out! The baby is coming out!” but still midwife N ignored her cry. Finally, a student nurse who was passing by conducted her delivery. (EP no. 20)*

### III. Unprioritized and disorganized nursing and midwifery management

One of the factors contributing to the disrespect for women was ‘*unprioritized and disorganized nursing and midwifery management*’ which was derived as a main category. Because both study sites had no concept of organized nursing and midwifery management, the midwives lacked accountability for their practice. This main category was supported by two subcategories: 1) lack of accountability and 2) unethical clinical practices.

#### Lack of accountability

The midwives’ practice was impromptu. They were not systematically assigned to a group of women, making their directive to attend to a delivery appear random. When there was a woman whose baby was coming out in front of the midwives, one of them would be directed to conduct the delivery.

*More than 30 women were in the crowded antenatal ward. The midwives had been sitting at the nurses’ station overviewing all the beds, but no midwives were checking on the women. At some point, a woman’s cry was heard and she was standing with her legs planted far apart. The other midwives prompted midwife J to go check on the woman and she slowly went to the woman. Upon arriving, the fetal head was already crowning and coming out. While midwife J was putting on her gloves, the baby came out and fell onto the floor. The baby died shortly afterward. (EP no. 21)*

*A staff member working at the antenatal ward brought a woman to the labor ward and left her there without informing the labor ward midwives. Midwife I who was at the labor ward nurse station heard the woman yelling and she went to see her. She looked all over for the woman and finally found her lying on the delivery bed. She quickly examined her cervical dilation without checking her chart and directly conducted the delivery without sufficient background information. (EP no. 23)*

#### Unethical clinical practices

In addition to the lack of accountability, there were no rule-based recordings or ethical charting. Many midwives made false reports by recording what they should have done but actually did not implement.

*After conducting one delivery, midwife D went back to the nurses’ station to complete the woman’s chart. She started to graph a point on the partograph even though nothing was written during the labor and delivery. Despite the fact that she had never checked the fetal heartbeat, woman’s vital signs, uterine contractions, or cervical dilatations, she falsified the information and the graph as well as faked the postnatal check-up, which was prior to the actual event. (EP no. 24)*

## Discussion

This study is one of the first few investigations that focused on direct observations of the actual behaviors of midwives in labor wards of two hospitals in urban Tanzania from the perspectives of respect and disrespect of women. The results vividly showed both respectful and disrespectful care, including some practices of midwives that women would not have realized as harmful such as ignoring the dose regulation of oxytocin. A synthesis of the observations and results also readily derives *weak nursing and midwifery management* as one of the contributors to disrespect for women.

### Positive interaction and assuring women’s rights

In previous studies, having positive interpersonal relationships between women and midwives in the forms of greeting, talking gently and patiently, creating an atmosphere where women can relax and feel comfortable, encouraging women, and explaining about the labor process and treatment, were reported as common aspects of respectful childbirth care [11, 22–25]. The White Ribbon Alliance (WRA) [11] stated seven corresponding women’s rights of childbirth as shown in Table 1. In the present study, four of those rights were protected by midwives’ respectful care. A minority of the midwives in the present study attempted to develop and maintain good relationships with the women by having conversational interactions and supporting them emotionally. According to several international guidelines of respectful childbirth care [11, 26, 27], women have the right to 1) be protected including their privacy in labor and delivery, 2) receive skin-to-skin mother-baby care and breastfeeding, and 3) receive continuous evidenced-based care throughout the childbirth process. The present findings indicate that a minority of midwives also attempted to consider women’s rights such as respecting women’s privacy and encouraging the mother-baby relationship. These minority of midwives also attempted to implement safe and timely care and treatment without ignoring their performance of a safe delivery by assessing the progress of labor and predicting delivery outcomes. In addition, they carefully observed safety and human rights during childbirth similarly to previous studies. However, as physical harm and harsh treatment were observed in the present study, the women’s rights to be free from harm and ill treatment [11] were not completely protected. Moreover, the women’s rights to be treated equally and free from discrimination, as well as to have liberty, autonomy, self-determination, and freedom from coercion [11] were not observed in the present study. To this end, additional observational studies are needed to determine if the midwives selectively disrespected some women and not others, and what factors were involved in such behavior.

**Table 1:** Comparison of respect and disrespect typologies

Bowser & Hill (2010)	Bohren et al. (2015)	White Ribbon Alliance (2011)	Present study	
			Respect for women	Disrespect for women
Physical abuse	Physical abuse - Use of force - Physical restraint	Freedom from harm and ill treatment		Physical abuse - Physical force including slapping, beating, pushing the abdomen in a non-emergency case; episiotomy without anesthesia; and harmful, unsanitary, and an unprofessional medical procedure
	Sexual abuse			
Non-consented care	Failure to meet professional standards ① - Lack of informed consent and confidentiality	Right to information, informed consent and refusal, and respect for choices and preferences, including the right to companionship of choice wherever possible	Positive interaction among midwives and women - Giving consideration to women’s emotions, greeting, implementing good communication skills, explaining what they were going to do, providing the results, and giving their own assessment and advice	Non-consented care - Performing medical treatment or physical examination without any explanation or consent from the women
Non-confidential care	Failure to meet professional standards ② - Physical examinations and procedures	Confidentiality, privacy	Respect for women’s privacy - Considering and protecting woman’s privacy from other women	Non-confidential care - Invading women’s physical and psychological privacy without any partition and asking women their private or personal information in front of others
Non-dignified care (including verbal abuse)	Verbal abuse - Harsh language - Threats and blaming	Dignity, respect	Positive interaction among midwives and women - Giving consideration to women’s emotions, greeting, implementing good communication skills, explaining what they were going to do, providing the results, and giving their own assessment and advice	Psychological abuse - Actions with violent words or harsh tones such as scolding, threatening, berating, and blaming, and emotional neglect by not genuinely sympathizing or considering the women’s situation
Abandonment or denial of care	Failure to meet professional standards ③ - Neglect and abandonment	Right to timely healthcare and to the highest attainable level of health	Provide safe and timely midwifery care for delivery - Periodic monitoring of the laboring process, performing appropriate care for delivery with precise timing by judging women’s laboring process Actively engage in women’s laboring process - Correcting both subjective and objective data to grasp and assess the progress of labor by going to the women’s side to predict what would be expected for the women’s childbirth	Abandonment of care - Ignoring, neglecting, and abandoning women during childbirth even if the women were yelling or screaming for help
Discrimination based on specific attributes	Stigma and discrimination - Discrimination based on sociodemographic characteristics - Discrimination based on medical conditions	Equality, freedom from discrimination, equitable care		
Detention in facilities				
		Liberty, autonomy, self-determination, and freedom from coercion		
			Encourage mother-baby relationship - Encouraging the relationship between the mother and the baby after giving birth by having skin-to-skin mother-baby care and breastfeeding	
	Poor rapport between women and providers - Ineffective communication - Lack of supportive care - Loss of autonomy			Psychological abuse - Actions with violent words or harsh tones such as scolding, threatening, berating, and blaming, and emotional neglect by not genuinely sympathizing or considering the women’s situation
				Unprioritized and disorganized nursing and midwifery management
	Health system conditions and constraints - Lack of resources - Lack of policies - Facility culture			Lack of accountability - One of the factors contributing to disrespect for women not systematically assigned to a group of women; the midwives’ practice was impromptu Unethical clinical practices - There were no rule-based recordings or ethical charting

### Expanded perspective of disrespectful care

According to previous studies focused on women’s experience of childbirth care in Tanzania, approximately 20% of postpartum women reported some form of physical or psychological D&A during childbirth such as being neglected and giving birth alone, being shouted at, receiving negative or threatening comments, and getting slapped or pinched [8, 12, 13]. Our observations of the practice of midwives in the present study also revealed the existence of maltreatment, and almost all of the D&A categories reflected previous reports. In the present study, we report five categories of disrespect that reflected the previous seven categories reported by Bowser & Hill’s [4] (See Table 1). However, we also included several new abusive and appalling behaviors.

Previous studies have identified various forms of physical abuse that included some kind of force such as slapping, beating, pushing the abdomen in a non-emergency case, and performing episiotomy without anesthesia [4, 6]. In the present study, several forms of physical abuse were observed which the women would not have thought of reporting to the researchers. The practices of the midwives were physically abusive and considered malpractice. These included the artificial rupture of the membrane using a contaminated fragment of a broken glass ampule, which is obviously harmful to the vagina, vulva, and fetal head, and facilitates the introduction of bacteria. Moreover, it is an unprofessional practice. On the part of the women, they may have seen it as just a necessary medical procedure and therefore they would not have reported it. However, this should be recognized as an abusive practice in terms of compromising safety. The incorrect use of oxytocic drugs by midwives also endangers the lives of women and their fetuses, although women would not be aware that such practice was physical abuse. Physical abuse, which is considered a malpractice, could be regarded as one of the WHO’s categorized D&A behaviors that women were unaware of [28].

Psychological abuse is a category similar to the previously categorized verbal abuse or nondignified care in previous studies. This form of abuse includes actions with violent words or harsh tones such as scolding, threatening, berating, and blaming [4, 6]. Our data revealed emotional neglect as a new dimension of psychological abuse. In this form of abuse, there is lack of soothing words for a suffering woman and failure to offer empathic words or actions for a woman whose baby just died. These behaviors similarly fall into the following mistreatment category of Bohren et al.: poor rapport between women and providers including lack of supportive care [6]. Although these psychological abuses may not appear to aggressively injure and bruise the women’s feeling, these abuses revealed that the midwives acted without empathy. This was reflected by their not offering a word of encouragement during labor pain or sympathy when the women lost their babies. Thus, not only using abusive language but also not providing emotional support to women can also be considered a form of psychological abuse. Women can usually face their own deliveries from a positive perspective with the strong support of midwives. Without this kind of support, women’s negative experiences towards childbirth may increase.

### Lack of professional accountability in midwifery practice

A contributing factor to the disrespect for women identified in previous studies was also identified in the present study. This category named ‘*lack of professional accountability in midwifery practice and no duty assignment*’ reflected the disorganized and dysfunctional nursing and midwifery management, facility culture, or work overload, rather than the midwives’ lack of ethical behaviors. This situation may reflect a broader picture of the problem in Tanzania. In previous studies, health system factors such as system deficiencies, unresponsive management, and health system conditions and constraints, were identified as contributors of D&A [6, 28–30]. Specifically, in the observed cases, midwives were not assigned to care for individual women and therefore they did not assume responsibility for monitoring their labor and delivery. Thus, it is possible that no one was assessing the labor progression of the individual women. This implies that the midwives might not have considered the assessment of labor progression as part of their responsibility, and they possibly expected other midwives to take care of the women. Thus, the deliveries were haphazardly and randomly conducted. Other contributors or drivers to D&A were also found to include facility and work-related factors such as heavy workloads, weak supportive supervision, and poor relations with co-workers [4–6, 31]. This category is equivalent to the structural disrespect and abuse as defined by Freedman et al. [28]. This involves systematic deficiencies that create a disrespectful or abusive environment such as an overcrowded and understaffed maternity ward where women deliver on the floor, alone, or in unhygienic conditions. Also, this category is similar to the health system factors of mistreatment: health system conditions and constraints described by Bohren et al. [6].

### Limitations of the study

This study has some limitations. The observations were conducted in only two hospitals and therefore possible bias could have been introduced. Being observed might have altered the midwives’ behaviors toward social desirability. However, this is doubtful given their abusive acts. Furthermore, there may have been some recall bias because the observer did not record the events as they happened but attempted to commit events to memory. The poster announcing our research may have affected the women’s behavior to the midwives. However, there were no comments from the midwives indicating that the researcher’s presence made a difference in the women’s behavior. Finally, the observations of midwives were conducted only during the day shift; the practices of the midwives during the night could have been different because of situational variables. However, the strength of the present study is that the behaviors of midwives were directly observed from the perspective of an experienced midwife with advanced degrees and multicultural experience. This is one of the rare studies that provides valuable data on direct observations of actual childbirth practices of midwives in urban Tanzania and the care received by women during childbirth.

A crucial aspect of D&A studies is the direct observation of midwifery behavior. Direct observation provides a rich source of data. However, the present researcher, who was a foreign non-licensed midwife in Tanzania, was in a difficult legal and moral position to intervene when faced with a dangerous abusive care requiring accurate interpretation and immediate decision. In retrospect, it might have been more prudent to discuss such potential issues with the health and research institution before conducting the observations. However, it was difficult to imagine the occurrences of such devastating abuses before the start of the study.

### Implications for practice and research

Nurses and midwives play a critical role in providing quality care during childbirth [32, 33]. Midwives who respect women and act professionally during childbirth are indispensable. Therefore, a midwifery educational system must have effective programs that raise awareness of D&A and teach respectful childbirth care. Health facility level factors that promote disrespectful behaviors must be identified and addressed. Jewkes & Penn-Kekana [34] stated that it is necessary to support institutions through resource allocation, training and supervision, and enforcement without blaming individual healthcare providers. To improve poor working conditions, it is necessary to streamline the complicated web of various systems, regulations, health policies, and budget allocations by close cooperation and collaboration among researchers, key health program planners, and the Tanzanian government.

## Conclusions

Both respectful care and disrespectful care of women during childbirth given by midwives were directly observed from health facilities in urban Tanzania. In terms of respectful care, the midwives often delivered care within the context of the women’s human rights. They developed and maintained a good relationship with the women by having positive verbal interactions, offering emotional support, and providing timely care for safe deliveries. In terms of disrespectful care, there were many disrespectful care and appalling practices during childbirth by the midwives in the forms of physical and psychological abuse, non-confidential care, non-consented care, and abandonment of care. Some types of physical and psychological abuse had never been observed or previously reported. A closer assessment of possible factors contributing to the disrespectful care indicated the lack of accountability of the midwives as professionals resulting from weak nursing and midwifery management. To promote respectful care of women during childbirth, pre-service and in-service midwife trainings, improvements of working and environmental conditions, and streamlining of various systems by close cooperation and collaboration between researchers, health institutions, and the Tanzanian government are needed. Empowerment of women is also necessary to ensure normal delivery.

## Abbreviations

AMReC: Asia Africa Midwifery Research Center
D&A: Disrespect and Abuse
NIMR: National Institute for Medical Research
WRA: White Ribbon Alliance
